# Updates on the Management of Non-Melanoma Skin Cancer (NMSC)

**DOI:** 10.3390/healthcare5040082

**Published:** 2017-11-01

**Authors:** Artur Fahradyan, Anna C. Howell, Erik M. Wolfswinkel, Michaela Tsuha, Parthiv Sheth, Alex K. Wong

**Affiliations:** 1 Division of Plastic and Reconstructive Surgery, Keck School of Medicine, University of Southern California, Los Angeles, CA 90033, USA; afahradyan@chla.usc.edu (A.F.); ahowellc@gmail.com (A.C.H.); erik.wolfswinkel@med.usc.edu (E.M.W.); 2 Division of Plastic and Maxillofacial Surgery, Children’s Hospital of Los Angeles, Los Angeles, CA 90027, USA; mtsuha@chla.usc.edu; 3 Keck School of Medicine of University of Southern California, Los Angeles, CA 91001, USA; shethp@usc.edu

**Keywords:** non-melanoma skin cancer, basal cell carcinoma, cutaneous squamous cell carcinoma, management of non-melanoma skin cancer, surgical margins of non-melanoma skin cancer, sentinel lymph node biopsy in non-melanoma skin cancer

## Abstract

Non-melanoma skin cancers (NMSCs) are the most common malignancy worldwide, of which 99% are basal cell carcinomas (BCCs) and squamous cell carcinomas (SCCs) of skin. NMSCs are generally considered a curable diseases, yet they currently pose an increasing global healthcare problem due to rising incidence. This has led to a shift in emphasis on prevention of NMSCs with development of various skin cancer prevention programs worldwide. This article aims to summarize the most recent changes and advances made in NMSC management with a focus on prevention, screening, diagnosis, and staging.

## 1. Introduction

Non-melanoma skin cancers (NMSC), also known as keratinocyte cancers, are the most common malignancy worldwide [1,2]. The incidence rate is the highest in Australia with more than 1000 per 100,000 person-years, followed by Europe with 98 per 100,000 person-years [1,2,3,4]. The number of people with NMSC increased from 2.4 million to 3.3 million from 2006 to 2012 in the USA [5]. Basal cell carcinoma (BCC) and squamous cell carcinoma (SCC) of the skin make up 99% of all NMSC, with BCC being 3 to 5 times more common than SCC [1,4,5,6,7]. Other much less common forms of NMSC include: Merkel cell carcinoma (MCC), primary cutaneous B-cell lymphoma, Kaposi sarcoma, carcinosarcoma, and dermatofibrosarcoma [6,8]. Although NMSCs are generally considered to be curable, they pose a vast problem for healthcare worldwide due to rising incidence [1,9,10,11]. Some NMSCs are associated with fatal outcomes. In particular, SCC is responsible for the majority of NMSC-related deaths [9]. The purpose of this paper is to discuss the updates on current management of NMSC and review the latest American Joint Committee on Cancers (AJCC) and National Comprehensive Cancer Network (NCCN) guidelines.

## 2. Brief Overview of Etiology, Risk Factors and Staging

### 2.1. Etiology and Risk Factors

The hypothesis that sunlight leads to skin cancer has existed for more than a century, however, the mathematical relationship between ultraviolet (UV) exposure and the risk of skin cancers was first described by Fears et al. in 1977 [12,13]. The authors suggested that while melanoma was primarily caused by intermittent sun exposure, keratinocyte cancers were related to cumulative doses of ultraviolent light [14,15]. Additional risk factors for development of NMSC include radiation therapy, lower Fitzpatrick skin types 1–4, prolonged immunosuppression, human immunodeficiency virus (HIV), human papilloma virus (HPV), and a diagnosis of certain syndromes or genetic disorders [16,17,18,19,20,21,22,23,24,25,26,27,28,29,30,31,32,33,34]. Unusual changes in peristomal skin of ileostomy and gastrostomy, burn scars, chronic inflammatory dermatologic conditions, and non-healing ulcers should also raise a suspicion for SCC, particularly Majolin’s ulcers [35,36,37,38,39,40,41,42]. Moreover, it has been well documented that male gender, tumor location in the trunk and extremities, superficial histologic subtype, younger age at first diagnosis of BCC, and red hair phenotype are associated with a higher risk of having multiple lesions [43,44,45,46,47,48].

Immunosuppressed patients have a particularly increased risk of developing NMSC when compared to the general population. Patients with HIV have a nearly 3-fold increased risk of developing primary NMSC as compared to the general population and a 44% increased risk of subsequent NMSC [49,50,51,52]. Additionally, the estimated standardized incidence rate for invasive NMSC is up to 82 times greater in patients who have received a kidney transplant when compared to non-transplanted patients [53]. Furthermore, immunosuppressed patients have an increased risk for rare types of NMSC, such as KS and MCC [54,55].

Lastly, the causal link between some risk factors and NMSC has been debated. Some evidence suggests a possible increased risk of NMSC in patients that are chronically exposed to certain photoreactive medications such as tetracycline, although further studies are needed to investigate these relationships [56]. Additionally, there are reported cases of NMSC occurring in the tattooed areas of the skin, but direct association between tattoos and skin cancers has not been established. As such, the FDA does not consider NMSCs complications of tattoo application [57]. Nevertheless, some still suggest that skin cancers should be included in the list of potential complications of tattooing given the actual incidence may be underreported [58].

### 2.2. Staging

BCCs rarely require staging given their minimal potential for metastasis. However, cutaneous SCC has a 4% annual incidence of metastasis, so staging is vital to its management and treatment [59,60,61]. The staging system of SCC is continuously being updated to meet current data. The staging system published in 2010 by the American Joint Committee on Cancers (AJCC) and the International Union Against Cancer (UICC) had some limitations. For example, T3/T4 tumors were reserved for bony invasion, which was very rare at 0.3–3.0%, while the rest were T1/T2 [62]. Thus, 70–86% of poor outcomes occurred in patients with T1/T2 tumors resulting in heterogeneous outcomes [62,63,64]. Whereas, in Brigham and Women’s Hospital (BWH) tumor staging, 5% of tumors were high stage but they accounted for 60% of poor outcomes, indicating superior homogeneity [62]. In 2017, the AJCC published the 8th edition of the Cancer Staging Manual. The staging of cutaneous SCC was revised to reflect recent evidence concerning high-risk clinicopathologic features to improve the overall staging system, which can be seen in Table 1, Table 2, Table 3 and Table 4 [63,65]. It should be noted that because the majority of SCCs of skin occur in the head and neck area, both of the AJCC’s 7th and 8th guidelines apply to tumors from the head and neck [63,64,65,66].

## 3. Prevention of NMSC

The incidence of NMSC has been increasing worldwide [1,2,3,4,67]. The rising incidence has been associated with several factors, including raised awareness in the general population and among physicians, an increased number of patients undergoing surgical treatment with confirmed histopathology instead of cryotherapy or electrodessication, improved registration, an aging population, and increased exposure to ultraviolent (UV) radiation [13,14,15,68]. These have led to the development of various skin cancer prevention measures and programs worldwide, highlighting the importance of skin cancer prevention.

Prevention strategies are divided into primary and secondary strategies. Primary prevention measures target behavioral changes to reduce sun exposure, reinforce the use of adequate sun protection and discourage intensive tanning, while the secondary measures facilitate early detection [4,69]. Public education is one of the key aspects of primary prevention, which is aimed to increase group-oriented awareness. It is known that among teen girls, correlating sun damage with premature wrinkling is an effective way to raise awareness [10,70]. In teen boys, emphasizing the protection of painful burns rather than the long-term risks of NMSC is more efficient [10,70]. Introduction of sun protection policy guidelines for schools and implementation of mass media campaigns of public education may also have significant impact on behavioral modifications [71,72]. Increasing evidence suggests that some pharmacologic agents may also be effective in prevention of NMSC. There is now strong evidence that vitamin D plays an important role in photocarcinogenesis and progression of NMSC. Hence, pharmacologic modulation by vitamin D, 1,25(OH)2D3 and its analogs represents a promising new strategy for prevention of NMSC [73]. Nicotinamide (Vitamin B3) is another agent that has been proven to decrease the incidence of NMSC, most likely by its ability to enhance DNA repair, modulate the inflammation produced by UVR and reduce UV induced immunosuppression [74,75]. Preventing and treating actinic keratosis (AKs) with minimal side effects and a low cost may be a promising alternative for skin cancer prevention in immunosuppressed patients [76].

Secondary prevention strategies include screening of high-risk populations, skin self-examination and physician surveillance, which aim to reduce morbidity and mortality by detecting cancer in its early stages [77].

## 4. Diagnosis and Types of Biopsy Techniques

The gold standard for diagnosing NMSC includes a thorough physical examination followed by conventional biopsy of the lesion for histopathologic examination [78,79]. A thorough lymph node exam is essential for diagnosis of SCC due to the risk for metastasis. Patients at high-risk for developing NMSC require special attention, particularly for individuals who work outside and thus have chronic exposure to UV radiation, as well as immunocompromised individuals. Some features, such as vaguely margined, white indurated, scar-like plaques, or the recently described “candle wax” sign may indicate an aggressive form of BCC [80,81,82,83].

When a suspicious lesion is identified, the diagnosis needs be confirmed with histopathologic analysis, which requires invasive intervention to obtain a tissue sample. There are several non-invasive medical technologies available that may be used to decide what lesions need to be biopsied as to potentially avoid unnecessary invasive procedures. Such technology includes: dermoscopy, confocal microscopy, cross-polarized light and fluorescence photography, as well as optical coherence tomography with high-frequency ultrasound [78]. There are also newer options, such as multiphoton microscopy and Raman spectroscopy that are emerging as state-of-the-art tools [78]. These adjunct screening tools may be used to assess skin lesions to find characteristic features of NMSC before proceeding with formal biopsy. The use of dermoscopy in the US is increasing with a recent survey showing up to 81% of US dermatologists using dermoscopy [84]. While it was mainly used for differentiation of melanocytic lesions, it is now widely utilized to screen superficial BCC and SCCs as well [85,86,87]. Most of these diagnostic modalities require additional training, which is one of the limiting factors for their use in clinic. Digitally stained multimodal confocal mosaic imaging was recently compared with standard frozen histopathologic specimens, thus showing 90% sensitivity and 79% specificity before training and 99% sensitivity and 93% specificity after training [88]. The longer processing time and the lack of reimbursement by the insurance companies are other limiting factors [89]. However, with the conduction of more research to justify their use, assignment of reimbursement values, development of more training courses for dermatologists, incorporating them into residency programs and continued improvement in speed and accuracy of these techniques will allow physicians to overcome some of the barriers and potentially increase the role of these technologies in the management of NMSC [89].

### 4.1. Biopsy Techniques

Despite tremendous advances in medical care and the shift to evidence-based medicine over the past several decades, there are no specific diagnostic guidelines for biopsy techniques that are specific to the lesion type or characteristics for one of the most common cancers worldwide. There are several types of biopsy techniques that are commonly used in the diagnosis of NMSC, yet no technique has been deemed the gold standard. These techniques include: excisional, incisional, shave, and punch biopsy. Although punch and shave biopsies are the most commonly performed techniques for initial sampling of NMSC [90], no well-powered studies in current literature or guidelines exist to help the physician decide which biopsy technique should be employed. Most studies are retrospective reviews and are not able to provide evidence-based proof that one technique is superior over the others, but rather demonstrate that each technique has advantages and disadvantages. It has been shown that although the intent of a shave biopsy is to obtain a diagnostic sample, 15–40% of patients who underwent secondary excisions for BCC had no residual cancer [91,92,93,94]. While some argue that those 15–40% of patients may have undergone unnecessary secondary procedures, final histopathologic examinations may be inaccurate. Kimyai-Asadi and Goldberg showed that bread-loafing at 4 mm intervals of elliptical excision specimens from facial basal cell carcinomas excised with 2 mm surgical margins is only 44% sensitive in detecting residual tumor at the surgical margins [95]. Another study demonstrated that 33% of cases with the initial biopsy results of actinic keratosis had additional findings on deep sections, including 13% SCC, 4% BCC, and 3% invasive SCC [96]. In the same context, a recent study brings up another critical point while questioning the value of reporting margin status in biopsies of NMSC. Based on their findings, 23% of cases with negative margins on initial biopsy demonstrated positive margins upon deeper-level examination [97] to further highlight the significance of proper interpretation of biopsy results when formulating a treatment plan.

With technology emerging in all aspects of the healthcare field, many look for new solutions to NMSC diagnosis. A recent study suggests that the reflectance confocal microscopy (RCM) may have a comparable accuracy to punch biopsy with regards to diagnosing and subtyping BCC [96,98,99]. It may also have a role in monitoring nonsurgical skin cancer therapies or assessment of tumor margins prior to surgery or intraoperatively [100,101,102,103].

In summary, there are currently no guidelines available to direct the biopsy techniques for the diagnosis of NMSCs. As a result, the clinician may decide what technique is the most appropriate in each scenario by considering not only the characteristics of the lesions, but also the intent of the biopsy. We are however hopeful that this issue will be addressed soon as the American Academy of Dermatology plans on releasing such guidelines in 2017 [104,105].

### 4.2. Low-Risk versus High-Risk Lesions

The current treatment of NMSC is driven by a risk assessment of the lesions. These assessments are based on the recurrence rates for BCC and recurrence and metastasis for SCC. According to NCCN 2017 guidelines, NMSC are classified as low- and high-risk lesions [106]. The lesions that are located on the trunk and extremities, excluding pretibia, hands, feet, nails, ankles, and are less than 20 mm in size are considered low-risk, whereas greater than 20 mm lesions are considered high-risk lesions. If the lesions are located on the cheeks, forehead, scalp, neck, and pretibia, the cutoff for size is 10 mm between the high and low-risk lesions. Other factors that differentiate the low and high-risk lesions include: well defined versus poorly defined borders, primary versus recurrent lesions, immunocompetent versus immunosuppressed, no history or history of RT at lesion site and no perineural involvement. Histologic subtypes also affect the recurrence and/or metastatic risk of the lesions such as nodular superficial BCCs and well or moderately differentiated SCCs are considered low-risk, whereas aggressive growth patterns for BCCs and poor differentiation for SCCs on histologic examination are indicative for high-risk lesions; see Table 5 for details [106].

RT-radiation therapy, L-trunk and extremities (excluding pretibia, hands, feet, nail units, and ankles), M-cheeks, forehead, scalp, neck, pretibia, H-central face, eyelids, eyebrows, periorbital, nose, lips, chin, mandible, preauricular and post auricular skin/sulci, temple, ear, genitalia, hands, feet.

### 4.3. The Role of Imaging

The majority of NMSCs can be successfully managed without imaging. Nevertheless, some SCCs may be locally invasive and have the potential for distant metastasis, while large or aggressive BCCs can invade surrounding anatomic structures. Therefore, high-risk tumors may require radiologic imaging for optimal management (Table 6). When there is concern for bony invasion, computed tomography (CT) should be employed [107,108]. Likewise, if there is concern for soft tissue or perineural involvement, magnetic resonance imaging (MRI) should be performed [107,108].

## 5. Treatment of NMSC: Overview of Surgical and Non-Surgical Treatment Modalities; NCCN Guidelines and Surgical Margins

NMSCs are considered curable diseases and the treatment mostly comprises of lesion removal. In addition, systemic and topical pharmacotherapy, cryotherapy (CT), photodynamic therapy (PDT), laser, and radiotherapy (RT) are also used [109,110,111,112,113]. A variety of techniques may be employed when removing NMSC. Common techniques include tangential shave removal, curettage, electrodessication, Mohs micrographic surgery (MMS), and standard surgical excision [114,115]. Many factors come into play when deciding treatment strategies including the patients’ overall condition and the goals of care. The current literature suggests that the treating physician’s specialty could also affect the technique utilized. For example, while some dermatology articles suggest that tangential shave removal of certain lesions provides an acceptable cure rate, Stewart et al. found an unacceptably high rate of residual BCC and SCC after shave biopsies [114,116]. This reflects the controversy surrounding type of biopsy employed previously in this article.

Concerns surrounding therapeutic non-surgical NMSC removal is detailed by Garcia-Cazana et al.; one of the main concerns of non-surgical treatments has been the development of resistance through a variety of mechanisms since these modalities usually require multiple administrations [117,118]. Despite the wide range of non-surgical treatment modalities that currently exist, surgery remains the mainstay of treatment for the majority of patients with NMSC and offers the highest cure rate [119,120].

### 5.1. Surgical Approach: Mohs Micrographic Surgery vs. Surgical Excision

It is generally agreed upon that surgical removal of NMSC offers the best overall outcome. A recent meta-analysis comparing the different treatment modalities for NMSC demonstrated that surgical excision had superior outcomes when compared to cryotherapy, photodynamic therapy, radiotherapy, 5-flurouracil (5-FU), and imiquimod in terms of complete lesion response, clearance of NMSC, and cumulative recurrence probabilities [109]. Additionally, Drew et al. compared MMS with non-surgical therapies (imiquimod, FU, ingenol mebutate, cryotherapy) for superficial BCC and SCCis, which demonstrated that both MMS and non-surgical therapies had high 5-year recurrence free survivals with MMS having a small advantage. Moreover, more patients would prefer to undergo MMS again due to the prolonged treatment course and pain associated with non-MMS treatments [11].

In a systemic review by Bath-Hextall et al., surgical excision was compared to MMS and demonstrated no difference in 30-month recurrence rates in the treatment of BCC [121]. Similarly, no difference was found in the recurrence risk following treatment of recurrent BCC removed by surgical excision versus MMS [122]. A recent meta-analysis by Lansbury et al. indicated similar results for cutaneous SCC, signifying no difference between recurrence risk of surgical excision and MMS [123]. Ghareeb et al. recently published an article comparing the outcome of MMS versus surgical excision for less common skin cancers in 15,121 patients identified through the National Cancer Data Base [124]. They showed only 8% were treated with MMS, however, the odds of achieving negative margins was 3.15 times higher in patients undergoing MMS when compared to all of the other surgical procedures combined when controlled for tumor size, location and histology. When MMS was compared with four different surgical techniques individually, it was still superior to primary excision and biopsy followed by narrow excision, however, no statistically significant difference was identified when comparing MMS with wide excision and re-excision with >1 cm margins [124].

The anatomic location of the tumor may pose unique challenges in its surgical excision. It should be noted that head and neck skin cancers are particularly challenging to treat given the difficulty in determining the amount of tissue that should be excised to provide adequate cancer-free margins with the best functional and cosmetic outcome. Moncrief et al. showed that the common practice of intraoperative frozen section analysis (IFSA) of margins had unacceptably high false negatives for head and neck BCC (28.7%) and SCC (27.5%). This resulted in the abandonment of IFSA in their practice and support for intraoperative MMS, even in advanced cases [125]. In light of this, Walker et al. investigated the utility of quenched activity-based probe imaging to discriminate cancerous versus normal skin tissue for NMSC [126]. They validated the activation of the probe with hematoxylin-eosin-confirmed cancerous tissue with 0.989 and 0.894 sensitivity and specificity, respectively. However, further studies are needed to confirm if this new, rapid, and easy to interpret technology would provide actual cost-effective increased cure rates by reducing re-excision and recurrence rates [126].

Although surgical excision remains the most commonly performed procedure in the treatment of NMSC, adequate surgical margins have not yet been defined. In an attempt to address this issue, Shel et al. suggested surgical margins for low and high-risk BCC and SCC based on a retrospective review of margins on 495 lesions as defined by MMS [127]. They concluded that lesions located on the face are best treated with MMS but in situations when standard surgical excision is the only option, a minimum 5 mm margin for low-risk lesions and about a 1 cm margin for high-risk lesions are required to completely excise the lesions in 95% of cases [127].

NCCN currently recommends standard excision as a primary treatment choice for low-risk BCCs that can be excised with 4 mm clinical margins as well as low-risk SCCs that can be excised with 4–6 mm clinical margins [106]. In case of positive margins, MMS or resection with complete margin assessment or standard re-excision in the trunk and extremity area are recommended [106]. In high-risk BCCs and local high-risk SCCs, MMS or resection with a complete margin assessment is recommended. Standard excision with wider surgical margins with linear or delayed repair may also be performed for high-risk lesions; however, there are no specific recommendations for margin size, and if margins are positive, then MMS or resection with complete margin assessment can be performed [106].

### 5.2. Curettage and Electrodessication (C&E), Cryosurgery

Curettage and electrodessication (C&E) is a commonly performed procedure to remove various skin lesions by scraping off the lesion with a curate followed by cauterization. It can be used for low-risk BCC and SCC, but it is not recommended for high-risk lesions due to an unacceptably high recurrence rate of up to 27% [128,129,130,131,132,133]. The overall efficacy of the C&E has been shown to be dependent on physicians’ experience as well. Silverman et al. showed decreased average recurrence rate of BCCs from 17.0% to 7.3%, when comparing the rates following procedures performed in the years of 1955–1963 and 1973–1982 by the same group, which was the attributed to physicians’ experience, specifically increased thoroughness in the technique [134]. Curettage alone may be used for selected low-risk lesions and can have similar cure rates (96% 5-year cure rate) but better healing compared to C&E [130]. Overall, C&E is considered a reasonable treatment choice for small low-risk NMSCs. It is also the least expensive and fastest method among all treatment methods of NMSCs [130,131].

Cryotherapy may also be considered for small, low-risk BCCs and SCCs, and can be combined with curettage, but it is not recommended for high-risk lesions [132,133]. It is associated with low cost, yet given the high overall recurrence rate of 7.5% following the treatment of primary lesions and 13% for recurrent lesions, it is rarely utilized [135,136].

According to current NCCN guidelines, C&E is recommended as a primary treatment choice for low-risk BCCs and local low-risk SCCs excluding terminal hair-bearing areas, such as the scalp, pubic and axillary region, beard area in men, and if adipose tissue is reached [106].

### 5.3. Photodynamic Therapy (PDT)

Photodynamic therapy (PDT) utilizes light, oxygen, and photosensitizers. 5-Aminolevulinic acid (5-ALA) and methyl 5-aminolevulinate (MAL) are commonly used pro-drugs for PDT, which are administered locally or systemically and convert to protoporphyrin IX (PPIX), which is then further converted to heme inside the cells. However, the PPIX conversion to heme is usually a slower process which results in its accumulation in cancer cells. Upon light absorption, the PPIX then undergoes excitation, resulting in production of oxygen free radicals, ultimately leading to cancer cell death. [137,138,139,140,141,142,143,144,145,146,147,148,149,150,151,152,153,154,155,156,157,158,159,160]. PDT has shown to be effective in treating superficial BCC, SCCis, and actinic keratosis (AK) with excellent cosmesis, though, it is currently not recommended for invasive SCC and aggressive BCC subtypes such as basosquamous or morphoeic infiltrating types [110,111]. A recent retrospective review demonstrated excellent response of superficial BCC to MAL-PDT and thick BCC to meta-tetrahydroxyphenylchlorin-PDT (mTHPC-PDT), with a 95% complete response rate after one round of PDT, which was maintained at 3-year follow-up and slightly decreased to 92% at 5-year follow-up [161]. PDT has also proven effective in reducing the incidence of AKs and SCCis in patients with organ transplant on long-term immunosuppressive therapy [110,111]. A randomized, multi-center study of 81 organ transplant recipients with 881 NMSCs (mainly AKs) as compared MAL-PDT to standard treatment (curettage, cryotherapy, surgery, or laser) and demonstrated significantly fewer new AKs in the MAL-PDT treated area at 15 months post-treatment [143]. These results indicate that pre-treatment of “field cancerization” in immunosuppressed patients with PDT is a promising alternative in terms of skin cancer prevention in immunosuppressed patients.

Significant research is being conducted to enhance the efficacy of PDT to treat more advanced stages of skin cancers. A recently developed microneedle-assisted PDT is aimed to deliver targeted therapy, which would allow treatment of deeper lesions [112,144]. In a small case series, the combination of a 630 nm laser with intralesional 5-aminolevulinic acid allowed treatment of deeper lesions [145]. A recent clinical trial studying the administration of 5-FU as a neoadjuvant for 5-aminolevulinic acid-PDT (ALA-PDT) has shown increased tumor selective protoporphyrin IX levels and enhanced cell death in a mouse mode [146]. Another clinical trial has compared the efficacy of ALA-PDT with surgery versus surgery alone for SCC of all stages, demonstrating a lower recurrence risk in the experimental group (16.6% versus 30.0%, *p* < 0.005) [147]. New agents are also developed with higher photosensitizing efficacy and better pharmacokinetics to provide higher tumor uptake and potentially long-term tumor cure. Patel et al. developed a near infrared bacteriochlorin 3 and the corresponding stereoisomers to be used in PDT with higher photosensitizing efficacy and limited skin phototoxicity when compared to porphyrin-based PDT [148].

PDT has also been studied as a potential modality for the prevention of NMSC. Studies demonstrated an equal preventative effect of daylight-PDT (dl-PDT) and conventional-PDT (c-PDT) in high-risk patients; however, all of the patients reported a burning sensation and pain during c-PDT while dl-PDT was reported to be almost pain free, which is why dl-PDT is preferred [149,150,151]. The authors postulate that PDT can induce premature senescence and kill senescent cells induced by itself [151]. A large retrospective review in a high volume dermatologic clinic investigated the development of SCC at one year following the PDT treatment of 1404 patients with AKs and 45 with NMSCs [152]. They found that 11% of patients developed SCC, while the rest remained SCC free at 1-year follow-up. Factors associated with developing SCC were older age, SCC history, Fitzpatrick skin-type 1, and sixty-minute or less incubation time, which is the time period between the administration of photosensitizing agent and exposure of the skin area to the light source. Therefore, it was concluded that PDT may be more effective in younger patients and with greater than 60 min of incubation time [152]. In addition, PDT has a tolerable side effect profile and low cost, which makes it more appealing in the treatment and prevention of skin cancers [153]. The main side effect is pain, which increases in intensity with the number of sessions and if the lesions are located in the head and neck area [153].

### 5.4. Laser Therapy

Treatment of NMSC with lasers causes light absorption by blood vessels of the targeted area, resulting in thermal distraction that then leads to tumor regression [154,155]. This targeted vascular photothermal destruction preserves the normal surrounding area and with treatment of dermatologic conditions, can lead to excellent cosmesis [155,156].

There are four major laser types used in the treatment of skin cancers: solid-state, diode, dye, and gas lasers [157]. A recent review article by Soleymani et al. discusses the different types of laser treatments in detail [158]. This study demonstrated that vascular targeting pulse dye lasers (PDL) have shown promising results in different studies with a complete clinical response in up to 95% of patients; nevertheless, poor results were also seen. Most incomplete responders were large tumors. Lower energy and small spot size also contributed to a poor cure rate. The 595 nm wavelength lasers were superior to the 585 nm wavelength lasers in terms of treating BCC. They concluded that the 595 nm wavelength vascular-targeted lasers had promising clinical efficacy in the treatment of BCC; however, data for consistent long-term outcome is not yet available. CO_2_ lasers allow very thin tissue ablation in the range of 20 µm with each pass [159,160,161,162,163]. Several studies have shown a different efficacy of CO_2_ lasers for treatment of BCC with 85–100% cure rates, excellent cosmetic outcomes, and minimal complications [158]. Lasers have been less commonly used and studied for the treatment of SCC, with only a handful of studies demonstrating the efficacy of CO2 lasers in treatment of SCCis. In the largest study of CO2 laser therapy, 44 of the 48 patients with SCCis were treated with CO2 lasers and had a total clearance rate of 97.7% and a recurrence rate of 6.8% at a mean 18-month follow-up [164].

Laser therapies have also been used in combination with PDT and the results have been mixed. One study investigated the efficacy of MAL-PDT with and without ablative (CO2) laser for the treatment of microinvasive SCC [165]. They found a greater efficacy rate when treated with the PDT-laser combination when compared to PDT alone at three months (84.2% versus 52.4%, *p*-0.03), and the differences in efficacy remained significant at 24 months. They also found a significantly lower recurrence rate in the PDT-laser group compared to the PDT alone group (12.5% versus 63.6%, *p*-0.006) [165]. In a case series, all patients with solitary superficial BCC treated with a combination of fractional CO2 laser and PDT achieved pathologically confirmed resolution of BCC with no serious adverse effects [152]. PDT combined with 2940 nm ER:YAG (solid-state) laser treatment has also shown improved relative efficacy in a few studies, demonstrating a lower recurrence risk of BCC and Bowen disease as compared to laser alone therapy, however, the esthetic outcomes were superior in laser alone treatment patients and the patients seem to prefer it due to its simplicity [157,166,167,168,169] Other studies dispute the benefits of PDT-laser combined therapy. One study compared the efficacy of PDT combined with pulse dye laser (PDL) with PDT alone for the treatment of 62 BCCs in 15 patients [170] and found no difference in complete tumor regression between the two groups. Additionally, this study found that the recurrence rate was higher in the combined treatment group [170].

Diode lasers, also called near-infrared lasers, function through temperature increases of target tissue and kill tumor cells directly. They have shown some efficacy in the treatment of small superficial SCC [157].

In summary, laser therapy has demonstrated promising results in the treatment of superficial NMSCs; however, the overall efficacy remains inferior compared to more traditional treatment modalities. It appears that the most beneficial aspects of laser treatment are the minimal side effects and excellent cosmetic outcome. While its role may be expanded in the future for the treatment of NMSCs, its use is currently limited. More long-term follow-up studies are needed to validate their efficacy and determine possible indications of laser therapy in the management of NMSCs.

### 5.5. Radiation Therapy

Although radiation therapy has shown control rates of 75–100% in early stage BCC and SCC, the role of radiotherapy has significantly decreased in the treatment of NMSCs with the emergence of MMS [113,171,172,173,174,175]. Now, it is mainly recommended as a primary treatment method if surgery is contraindicated, if lesions are located in cosmetically sensitive areas, or as an adjunct to surgery in patients with advanced disease [113].

There are several techniques of radiotherapy, each having advantages and disadvantages. A recent survey study completed by 16 members of American Brachytherapy Society indicated that the majority of them prefer brachytherapy over external beam radiation therapy given the shorter treatment course, conformity of treatment for irregular or curved targets and shallow dose deposition [176]. Many of them routinely use ultrasound to estimate the depth and lateral extension of the lesion before initiating treatment, which has shown to be helpful for more accurate treatment planning [176,177]. The advantage of superficial radiotherapy (SRT) is that the maximum dose is at skin’s surface, and there is less penetration through eye shields, thus making it easier to use around the eye [178]. It has shown to provide 93–100% tumor control depending on the lesion size, whereas electron beam radiation provided 72–88% tumor control in the same study [179]. Another study demonstrated a recurrence risk of 5% at five years in patients with low-risk NMSCs that are treated with SRT [180]. Electron beam radiotherapy (EBRT) has the advantage of treating superficial lesions without causing significant damage to deeper structures and may be useful when treating skin cancers over bony prominences or cartilaginous structures [181]. Interstitial brachytherapy is another subtype that involves the insertion of a radioactive catheter within the tumor bed and is used to treat skin cancers in difficult areas such as eyelids [182].

Some of the common complications of radiation therapy include alopecia, pigmentary changes, telangiectasias, fibrosis, atrophy, buccal mucositis, gingivitis, tooth loss, and a loss of salivary gland function. In addition, more severe complications such as soft tissue or bone necrosis, cataracts, conjunctival scarring, or eyelid deformity may occur [113,183].

NCCN currently recommends radiotherapy as a primary treatment modality for non-surgical candidates in the treatment of low- and high-risk BCCs and local low- and high-risk SCCs (in high-risk SCCs, RT may be supplemented with chemotherapy) [106]. However, it is often reserved for patients over 60 years of age due to concerns for long-term sequelae. Conversely, it is contraindicated in patients with genetic conditions predisposing to skin cancer and connective tissue diseases [106]. RT is also recommended in high-risk lesions in cases of positive margins after surgical excision and/or if a negative margin is unachievable with MMS. It may also be considered if there is extensive perineural or large nerve involvement [106]. NCCN recommends RT with or without chemotherapy in patients with SCCs with positive lymph nodes on FNA or core biopsy who are not surgical candidates or possible adjuvant therapy following surgical resection of lesions [107].

### 5.6. Chemotherapy and Immunotherapy

Systemic chemotherapy has an important role in the management of advanced NMSC. The term “advanced NMSC” usually refers to metastatic disease and/or inoperable lesions due to size and location that would otherwise cause unacceptable functional impairment if treated with excision [21,113]. Platinum based preparations such as cisplatin, are the most commonly used chemotherapeutic agents for NMSC; however, other agents, including cyclophosphamide, bleomycin, doxorubicin, methotrexate, and 5-FU, may also be used [23,24,25,26,27,28,29,30,31,121,122,184,185,186,187,188,189]. A recent publication claims that monotherapy of patients with stage I and II lip SCC via superficial temporal artery administration of bleomycin derivative peplomycin is a highly effective treatment method for achieving a cure in 70.8% of patients [113]. It was reported though, that 12.5% of patients developed interstitial pneumonia, 12.5% had occurrence of metastasis, and 16.7% had local recurrence [113].

Certain immune modulators and monoclonal antibodies have demonstrated promising results. In this concept, epidermal growth factor receptor (EGFR) appears to be of particular interest. EGFR is involved in the pathogenesis of SCC [190], hence its inhibitors, such as cetuximab and panitumumab, may be used in the treatment of SCC [23]. In one study, cetuximab has shown 69% efficacy in disease control rate at 6 weeks when administered as a single agent in patients with advanced SCC [191]. In another study, 95% of patients became operable following administration of cetuximab combined with a chemotherapeutic agent [192]. Cetuximab’s reasonable tolerability makes it a potential agent for neo-adjuvant therapy [23]. In contrast, panitumumab did not show a superior efficacy in combination with chemotherapy over chemotherapy alone in CONSERT-1 phase II trial or in combination with radiotherapy over chemotherapy alone in CONSERT-2 phase II trial [193,194]. In both studies, grade 3, grade 4, and serious adverse effects were more common in the group receiving panitumumab, which makes the future of this agent questionable in the treatment of NMSCs.

NCCN currently recommends chemotherapy as a possible supplement to radiation therapy in local, high-risk SCCs for patients who are not surgical candidates [106]. It is also recommended as a primary treatment concurrent with RT in patients with FNA/core biopsy positive lymph nodes who have inoperable diseases [106].

### 5.7. The Hedgehog Pathway Inhibitors

The ability of some antifungal agents to inhibit the Hedgehog (HH) pathway has also been explored in the treatment of NMSC. While itraconazole has shown some efficacy in exploratory phase II trial, the toxicity remains a major limiting factor in terms of its use in clinical practice. Posaconazole is another antifungal with better safety profile, though further validation is still needed [23,195].

Vismodegib is a newer agent that acts as an inhibitor of the sonic hedgehog (SHH) pathway and was approved for adult patients with advanced BCC in 2012 [21,196]. Despite some controversial reports of Vismodegib being associated with development of new SCC, it still appears to have great interest among physicians; however, its significant adverse effects pose a challenge [21,197,198,199]. Sonidegib is another SHH inhibitor recently approved for treatment of advanced BCC in the United States of America (USA) with slightly better efficacy than Vismodegib [200]. They are both currently recommended by NCCN to be considered in high-risk BCCs if positive margins are present following surgical excision and/or negative margins are not achievable with MMS [106].

### 5.8. Topical Agents

Topical agents may be used to treat NMSC in instances when patients are not candidates for standard approach. Factors that may favor the use of topical pharmacotherapy include extensive, multifocal or multiple tumors, indistinct lesion boundaries, localization in cosmetically sensitive or difficult to treat areas, and a history of hypertrophic scars and keloid [201]. There are several topical agents that are currently used in the treatment of NMSC, some of which are approved by FDA. Topical 5-FU has been approved for treatment of AK and superficial BCC for many years, although its use for treatment of SCCis is off-label [202,203,204]. Some studies suggest a 90–96% cure rate of AK and superficial BCC with topical 5-FU [205,206]. Imiquimod is another agent that is approved by FDA for treatment of AK and superficial BCC, while diclofenac and ingenol mebutate are FDA approved for AK only [202]. Currently, there are many other topical agents still in the investigational stage, including: resiquimod, piroxicam, calcium dobesilate, and potassium dobesilate containing formulations, betulinin acid and topical retinoids that may have some efficacy for the treatment of skin cancers [202].

## 6. Utility of Sentinel Lymph Node Biopsy (SLNB) and Regional Lymphadenectomy

Sentinel lymph node biopsy (SLNB) is usually not considered for BCC given the very rare chance of metastasis. In contrast, SLNB may play a significant role in the management of SCC. An association of positive SNL and poor prognosis of cutaneous SCC has been previously demonstrated in several studies, showing a 96% 5-year survival with no regional lymph node involvement [206,207,208,209,210,211,212,213,214,215]. The 5-year survival decreases to 72% with adequate treatment, and 25–35% with no treatment [207,208,209,210,211,212,213,214,215,216]. A recent study showed that patients with no micrometastasis to SNL developed no local and distant disease at an average follow-up period of 27.5 months [217]. Another recently published report indicated that positive results were seen in 13.4% of 364 SNLBs in patients with cutaneous SCC, which is similar to that of melanoma [218]. They also found that positive SNL was associated with poor prognosis; hence their findings further support the utility of SLNB in the management of SCC. However, while it appears that consensus exists in terms of positive rate of SNL and poor prognosis associated with it [219,220], many still question the utility of SNLB due to inadequate evidence that supports it actually improves the prognosis the disease. Maryuma et al. showed that despite an 18.4% positive rate of micrometastasis to SNL in patients who underwent SNLB, there was no difference in terms of metastasis-free and disease-specific survival rates between the groups who did and did not undergo SNLB, regardless of T-staging [221]. Silberstein et al. investigated the rate of metastasis to lymph nodes in 572 patients with 725 head and neck cutaneous T1 and T2 SCC with 1.09% and 5.46% positive results, respectively, and recommended no SNLB for clinically N0 patients based on these findings [222]. While there is no indication for SLNB in the current NCCN guidelines, some authors still recommend it given that SNLB may potentially improve patients’ prognosis if enough data is available [106,217].

When palpable regional lymph nodes are present or abnormal lymph nodes are identified on imaging studies, FNA or core biopsy is warranted [106]. In the case of positive results, excision of primary tumor and proper nodal dissection followed by chemoradiation therapy is a standard of care; however, prophylactic regional nodal dissection in clinically N_0_ disease still remains controversial [106,222].

## 7. Conclusions

Despite the emergence of novel non-surgical treatment modalities, surgical resection remains the most common treatment method for NMSCs with 4 mm clinical margins in low-risk BCCs and 4–6 mm in local low-risk SCCs. However, NCCN offers no clear guidelines in terms of margins in high-risk lesions. In high-risk BCCs and local high-risk SCCs, MMS, or surgical resection with complete margin assessment is recommended. RT is usually utilized for local lesions if the patient is not a surgical candidate or in metastatic disease with or without chemotherapy. Several topical agents, such as 5-FU and imiquimod, are FDA approved for superficial BCCs and AKs, while diclofenac and ingenol mebutate are FDA approved for AKs only. However, the off-label use of topical agents for treatment of SCC is also reported. PDT and laser therapy are relatively newer treatment modalities for NMSCs, with a reasonable success rate in low-risk lesions, though, more research is required to study their long-term outcomes, which may allow for the expansion of their utility in the management of skin cancers. Finally, the sonic hedgehog pathway inhibition is another emerging area of the treatment NMSCs with vismodegib and sonidegib already being approved for high-risk BCCs.

There appears to be no consensus in terms of recommended skin biopsy techniques for definitive diagnosis, as well as the role of sentinel lymph node biopsy. Though sentinel lymph node biopsy is warranted in cases of palpable lymphadenopathy, current data on regional lymph node dissection are sparse and more research is needed for any recommendation.

## Figures and Tables

**Table 1:** American Joint Committee on Cancer Tumor (T) Classification, 8th Edition [65].

SCC	
T_X_	Primary tumor cannot be assessed
T_0_	No evidence of primary tumor
T_is_	Carcinoma in Situ
T_1_	Tumor < 2 cm in greatest dimension
T_2_	Tumor ≥ 2 cm and <4 cm in greatest dimension
T_3_	Tumor ≥ 4 cm in greatest dimension and/or perineal invasion and/or deep invasion and/or minor bone erosion
T_4a_	Tumor with gross cortical bone/marrow invasion
T_4b_	Tumor with skull base invasion and/or skull base foramen involvement

**Table 2:** American Joint Committee on Cancer Tumor (N) Classification, 8th Edition [65].

SCC	
Clinical N (cN)	
N_X_	Regional lymph nodes cannot be assessed
N_0_	No regional lymph node metastasis
N_1_	Metastasis in single ipsilateral lymph node, ≤3 cm in greatest dimension and ENE (−)
N_2a_	Metastasis in single ipsilateral lymph node, >3 cm but not >6 cm in greatest dimension and ENE (−)
N_2b_	Metastasis in multiple ipsilateral lymph node, none >6 cm in greatest dimension and ENE (−)
N_2c_	Metastasis in bilateral or contralateral lymph nodes, none >6 cm in greatest dimension and ENE (−)
N_3a_	Metastasis in a lymph node >6 cm in greatest dimension and ENE (−)
N_3b_	Metastasis in any node (s) and clinically overt ENE (+)
Pathologic N (pN)	
N_X_	Regional lymph nodes cannot be assessed
N_0_	No regional lymph node metastasis
N_1_	Metastasis in single ipsilateral lymph node, ≤3 cm in greatest dimension and ENE (−)
N_2a_	Metastasis in single ipsilateral lymph node, ≤3 cm in greatest dimension and ENE (+), or in single ipsilateral lymph node, >3 cm but not >6 cm in greatest dimension and ENE (−)
N_2b_	Metastasis in multiple ipsilateral lymph nodes, none >6 cm in greatest dimension and ENE (−)
N_2c_	Metastasis in bilateral or contralateral lymph, node (s), none >6 cm in greatest dimension and ENE (−)
N_3a_	Metastasis in a lymph node >6 cm in greatest dimension and ENE (−)
N_3b_	Metastasis in a single ipsilateral node >3 cm in greatest dimension and ENE (+); or multiple ipsilateral, contralateral, or bilateral nodes, any with ENE (+); or a single contralateral node ≤ 3 cm and ENE (+)

ENE–extranodal extension.

**Table 3:** American Joint Committee on Cancer Tumor (M) Classification, 8th Edition [65].

SCC	
M_X_	Distant metastasis cannot be assessed
M_0_	No distant metastasis
M_1_	Distant metastasis

**Table 4:** American Joint Committee on Cancer Tumor Staging, 8th Edition [65].

SCC			
Stage	T	N	M
0	T_is_	N_0_	M_0_
I	T_1_	N_0_	M_0_
II	T_2_	N_0_	M_0_
III	T_3_	N_0_ or N_1_	M_0_
IV	T_1_ or T_2_	N_1_	M_0_
	T_1_, T_2_ or T_3_	N_2_	M_0_
	Any T	N_3_	M_0_
	T_4_	Any N	M_0_
	Any T	Any N	M_1_

**Table 5:** National Comprehensive Cancer Network (NCCN) 2017 Guidelines for Risk Factors for Recurrence for basal cell carcinoma (BCC) and Recurrence and Metastasis for squamous cell carcinomas (SCC) [106].

	Low-Risk	High-Risk
BCC		
Location/size	L < 20 mm M < 10 mm	L > 20 mm M ≥ 10 mm H
Borders	Well Defined	Poorly Defined
Primary vs. Recurrent	Primary	Recurrent
Immunosuppression	(−)	(+)
Site of Prior RT	(−)	(+)
Pathology Subtype Perineural Involvement	Nodular Superficial (−)	Aggressive Growth Pattern (+)
SCC		
Location/size	L < 20 mm M < 10 mm	L > 20 mm M ≥ 10 mm H
Borders	Well Defined	Poorly Defined
Primary vs. Recurrent	Primary	Recurrent
Immunosuppression	(−)	(+)
Site of Prior RT or Chronic Inflammatory Process	(−)	(+)
Rapidly Growing Tumor	(−)	(+)
Neurologic Symptoms	(−)	(+)
Pathology Degree of Differentiation Adenoid, Adenosquamous, Desmoplastic, Metaplastic	Well or Moderately Differentiated (−)	Poorly Differentiated (+)
Depth, Thickness or Clark Level	<2 mm or I, II, III	≥2 mm or IV, V
Perineural, Lymphatic, or Vascular Involvement	(−)	(+)

**Table 6:** Indications for Radiologic Imaging for Skin Cancers and Recommended Imaging Studies [107,108].

Possible Bony Invasions	CT *
Possible Orbit Invasions	CT-bony invasion, MRI **-soft tissue
Assessment the extent of Tumor Invasion in Soft Tissue	MRI
Staging of Lymph Nodes and Metastatic Disease	CT or MRI or PET ***(PET-CT)
Evaluation for Potential Perineural Spread	MRI
Post-operative Surveillance for Recurrent Disease	PET-CT

* CT-computer tomography; ** MRI-magnetic resonance imaging; *** PET-positron emission tomography.
